# Evaluating temporal patterns of snakebite in Sri Lanka: the potential for higher snakebite burdens with climate change

**DOI:** 10.1093/ije/dyy188

**Published:** 2018-09-11

**Authors:** Dileepa Senajith Ediriweera, Peter John Diggle, Anuradhani Kasturiratne, Arunasalam Pathmeswaran, Nipul Kithsiri Gunawardena, Shaluka Francis Jayamanne, Geoffrey Kennedy Isbister, Andrew Dawson, David Griffith Lalloo, Hithanadura Janaka de Silva

**Affiliations:** 1 Centre for Health Informatics, Biostatistics and Epidemiology, Faculty of Medicine, University of Kelaniya, Ragama, Sri Lanka; 2 Centre for Health Informatics, Computing and Statistics, Lancaster University Medical School, Lancaster, UK; 3 Department of Public Health, Faculty of Medicine, University of Kelaniya, Ragama, Sri Lanka; 4 Department of Parasitology, University of Kelaniya, Ragama, Sri Lanka; 5 Department of Medicine, Faculty of Medicine, University of Kelaniya, Ragama, Sri Lanka; 6 South Asian Clinical Toxicology Research Collaboration, University of Peradeniya, Peradeniya, Sri Lanka; 7 Clinical Toxicology Research Group, University of Newcastle, Waratah, Australia; 8 Addiction Medicine, Central Clinical School, Faculty of Medicine, University of Sydney, Sydney, Australia; 9 Department of Clinical Sciences, Liverpool School of Tropical Medicine, Liverpool, UK

**Keywords:** Sri Lanka, snakebite, seasonal variation, weather, relative humidity, global climate change

## Abstract

**Background:**

Snakebite is a neglected tropical disease that has been overlooked by healthcare decision makers in many countries. Previous studies have reported seasonal variation in hospital admission rates due to snakebites in endemic countries including Sri Lanka, but seasonal patterns have not been investigated in detail.

**Methods:**

A national community-based survey was conducted during the period of August 2012 to June 2013. The survey used a multistage cluster design, sampled 165 665 individuals living in 44 136 households and recorded all recalled snakebite events that had occurred during the preceding year. Log-linear models were fitted to describe the expected number of snakebites occurring in each month, taking into account seasonal trends and weather conditions, and addressing the effects of variation in survey effort during the study and of recall bias amongst survey respondents.

**Results:**

Snakebite events showed a clear seasonal variation. Typically, snakebite incidence is highest during November–December followed by March–May and August, but this can vary between years due to variations in relative humidity, which is also a risk factor. Low relative-humidity levels are associated with high snakebite incidence. If current climate-change projections are correct, this could lead to an increase in the annual snakebite burden of 31.3% (95% confidence interval: 10.7–55.7) during the next 25–50 years.

**Conclusions:**

Snakebite in Sri Lanka shows seasonal variation. Additionally, more snakebites can be expected during periods of lower-than-expected humidity. Global climate change is likely to increase the incidence of snakebite in Sri Lanka.

**Key Messages**

- Snakebite incidence shows seasonal variation in Sri Lanka, with the highest incidence during November–December followed by March–May and August.
- Lower-than-expected relative humidity in a given season leads to higher snakebite incidence.
- Global climate change is likely to increase the snakebite burden in Sri Lanka during next 25–50 years.
- This study demonstrates a general approach to addressing the effects of variable survey effort and recall bias associated with epidemiological surveys.

## Introduction

Sri Lanka—a tropical island nation in the Indian ocean—is host to more than 100 terrestrial snake species. Annual snakebite incidence in Sri Lanka is about 400 per 100 000 people, corresponding to 80 000 snakebites in a 20 million population.^1^^,^^2^ Previous data have indicated geographical variation and seasonal patterns in snakebites across the world.^3–5^ Previous local studies have also reported seasonal variation in hospital admission rates due to snakebites, e.g. the peak incidence in admissions due to bites by *Bungarus caeruleus* in September–October^6^ and by *Daboia russelii* in March–April and October–November.^7^ Similar patterns have been reported in other parts of the world.^8–13^

There are four climate seasons in Sri Lanka; the South-West monsoon (‘Yala’ season) operates from May to September, bringing rain to central highlands, southern and western parts of the country, whilst the North-East monsoon (‘Maha’ season) lasts from December to February, bringing rain into the northern and eastern parts of the country. Sri Lanka is an agricultural country whose agricultural activities are mainly carried out in the ‘Yala’ and ‘Maha’ seasons.^14^^,^^15^ Snakebite is considered as an occupational hazard; high incidences of snakebites have been reported during the periods of high rains and agricultural activity.^3^^,^^6^^,^^7^^,^^16^

Global climate change is likely to have adverse effects on health during the twenty-first century, especially in developing countries with low income. Increasing incidences in a number of food-borne, water-borne and vector-borne diseases have been recognized, but snakebite, which is a disease of poverty, has not been emphasized.^17^^,^^18^ According to the representative concentration pathways’ (RCP) intermediate scenarios (i.e. RCP 4.5 and RCP 6),^19^ Sri Lanka will experience an increase of approximately 1.0–2.0°C in maximum temperature by 2100.^20^^,^^21^ Snakes are cold-blooded animals and climate change is likely to alter their geographical distribution. Previous studies have attempted to evaluate the effect of climate change on snakebite based on snake distributions and have highlighted the necessity for further research,^22–24^ but no studies have evaluated the effect of climate change on the epidemiology of snakebite.

Determining the geographical variation of snakebites provides useful information by identifying high-risk areas. This helps policy makers to target preventive measures and to allocate resources efficiently at the local level. Understanding the seasonal variation in snakebite incidence is also important to determine resource allocation at the operational level, e.g. the distribution of anti-venom based on seasonal requirements and deployment of targeted preventive measures.^25^^,^^26^ Geographical variation in snakebite incidence has been studied in Sri Lanka, but temporal snakebite patterns have not been evaluated. In this paper, we analyse the seasonal pattern of snakebite incidence and show the possible effect of global climate change on snakebite incidence in Sri Lanka during the next 25–50 years. Our methodology highlights a general approach to addressing the effects of variable survey effort and recall bias associated with epidemiological surveys of this kind.

## Methods

### Definitions

*Survey month* is the month where the survey was conducted. The National Snakebite Survey was conducted for 11 consecutive months and *survey months* are denoted by ‘*s*’ (i.e. *s* = 1, 2,  … 11).

*Bitten month* is the month where the victim experienced a snakebite. The National Snakebite Survey reported snakebite events that occurred during a period of 23 months and *bitten months* are denoted by ‘*t*’ (i.e. *t* = 1, 2, … 23). Note that the 13th *bitten month* corresponds to the 1st *survey month*, as survey reported snakebite data for the preceding 12 months.

Average meteorological parameters in a given bitten month represent the mean value across all the locations that reported snakebites in a given bitten month (e.g. average rainfall in January 2012 represents the mean rainfall over all the locations that reported a snakebite in January 2012).

### Data sources

#### Epidemiological data

An island-wide community-based National Snakebite Survey was conducted between August 2012 and June 2013.^1^ The study sampled about 1% of the Sri Lankan population. Sri Lanka has 9 provinces and 25 districts. The survey design used multistage cluster sampling, with 125 clusters sampled from each province and clusters allocated to districts within each province in proportion to the population size of each district. In each cluster, 40 consecutive households were sampled from a random initial household on the electoral register. In the event of non-response (nobody in the household after two visits), the next house on the electoral register was selected. Data were collected by conducting face-to-face interviews with an adult household member. All snakebite events occurring in the sampled households during the year preceding the date of survey were recorded along with the date of each snakebite.

#### Meteorological measurements of weather

Average meteorological measurements in each bitten month were obtained from the Department of Meteorology, Sri Lanka. Meteorological measurements included rainfall, minimum temperature, maximum temperature, minimum relative humidity and maximum relative humidity. Meteorological measurements (i.e. weather data) pertaining to each location and time (i.e. bitten location and month) were then attached to each individual bite report. We then calculated the average meteorological measurements for each bitten month as the average value reported in all the bitten locations in a particular bitten month and used these averaged meteorological values in each bitten month as explanatory variables for the analysis.

### Statistical methods

The National Snakebite Survey in Sri Lanka was conducted over 11 consecutive months. Any snakebite experienced by any household member within the year preceding the date of interview was recorded in the survey. Therefore, the survey included snakebite events occurring between August 2011 and June 2013 (i.e. 23 months). We call the month in which the survey was carried out the *survey month* and the month in which the snakebite occurred the *bitten month*. The number of snakebites recorded in each bitten month is given in Supplementary Figure 1, available as Supplementary data at *IJE* online.

#### Accounting for survey methodology

The number of snakebites recorded in each bitten month depends on both the risk of snakebite and the effective sample size for the given month. The effective sample size for a given month in turn depends on the survey effort in that month (i.e. number sampled in a given month) and the number of survey months that contributed snakebite records to a particular bitten month. Survey effort also varied over time due to the variation in population density and the extent of the geographical area surveyed in a particular month (Supplementary Figure 2, available as Supplementary data at *IJE* online). Note that more than 1 survey month can contribute snakebite records to a particular bitten month. For example, only the survey month August 2012 provided records of snakebites in the bitten month August 2011, whereas the August and September 2012 surveys both contributed to the number of snakebite records for the bitten month September 2011. For the data analysis, the number of snakebites recorded in each bitten month was further disaggregated across each of the contributing survey months so that the response variable was the number of bites recorded at each combination of bitten month and survey month, whilst survey effort in each survey month was considered as an offset. Our data include 22 snakebites that occurred during the period of 23 bitten months but with recall duration more than 12 months. Therefore, our study had 158 combinations of bitten and survey months (i.e. 11 bites occurring in the same survey month, 125 bites pertaining to the preceding 12 months and 22 bites that occurred more than 12 months before the survey month but within the 23 bitten months).

#### Accounting for recall bias

The survey recorded snakebite events that occurred throughout the year preceding the date of survey, so the number of recorded snakebites in any bitten month would be affected by recall bias. For this reason, the statistical model included the time difference between survey month and bitten month as a log-linear effect, corresponding to an exponentially decaying recall power.

#### Modelling snakebite events

The National Snakebite Survey reported 695 snakebite events from a sample of 165 665 individuals. Twenty snakebite events were removed during data cleaning due to missing values. We fitted a Poisson log-linear model to the number, *Y*_s__*t*_, of snakebites occurring in each bitten-survey month, with expected numbers of bites:

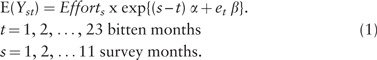


In Equation (1), *s* is the survey month, *t* is the bitten month, *e_t_* is the exposure vector in month *t* and *Effort_s_* is the number of people sampled in survey month *s* divided by the total population of Sri Lanka. The following terms were considered for inclusion in the exposure vector: bitten month *t*, corresponding to a log-linear trend over the study period; sine and cosine functions with periodicities of 12, 6, 4 and 3 months to investigate seasonal effects; average values of minimum temperature, maximum temperature, minimum relative humidity, maximum relative humidity and rainfall with time-lags of 0, 1, 2 and 3 months. All of the meteorological variables were de-seasonalized using sine and cosine functions to avoid confounding of weather effects with other, unrecorded seasonally varying factors. We refer to these de-seasonalized series as *weather anomalies.*

Forward variable selection with log-likelihood ratio assessment was used to determine which explanatory variables should be included in the exposure vector *e_t_*. Confidence intervals (CIs) were calculated using the standard Normal approximation to the sampling distribution of the maximum-likelihood estimates of the model parameters, and validated using a bootstrap method (Supplementary Material, available as Supplementary data at *IJE* online). Goodness of fit of the model was assessed by the ratio between residual deviance and residual degrees of freedom and by residual plots. All computations used the R programming language version 3.2.3.^27^

The expected numbers of reported snakebites in any month, after adjusting for recall bias for each bitten-survey month, were estimated from the fitted log-linear model and used to calculate monthly snakebite estimates and 95% CIs for the whole country. Monthly snakebite estimates were then decomposed into seasonal and weather-related components, and predictions made under future climate scenarios. The only weather-related explanatory variable included in the fitted log- linear model was relative humidity. We used the association between relative humidity and temperature to predict snakebite burden under scenarios corresponding to changes in temperature with rainfall fixed at current levels. Median monthly average levels of maximum relative humidity and maximum temperature over the study period were 89% and 31°C, respectively. We would expect approximately a 2.5% reduction in humidity if the temperature were to increase by 0.5°C, holding all other environmental conditions fixed.^28^ We therefore predicted the country-wide monthly seasonal snakebite incidence under this scenario, i.e. for an average relative-humidity anomaly of –2.5%, to reflect a 0.5°C increase in the mean annual temperature in the country.

### Ethics statement

Ethical approval for the National Snakebite Survey was obtained from the Ethics Review Committee of the Faculty of Medicine, University of Kelaniya. All interviews were conducted after obtaining informed written consent. Permission for conducting the survey was obtained from District- and Divisional-level public administrators.

## Results

### Weather patterns

**Figure 1.**
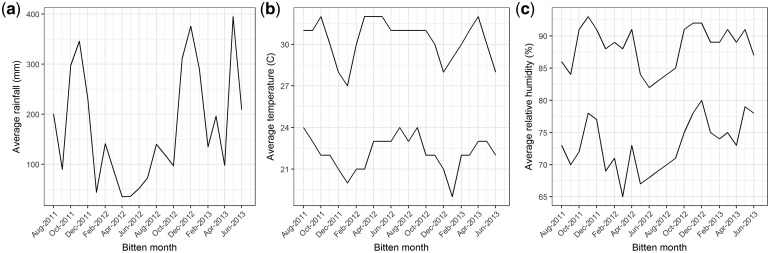
(**a**) Average rainfall; (**b**) average minimum temperature and maximum temperature; (**c**) average minimum relative humidity and maximum relative humidity in each bitten month.

Rainfall, relative humidity and temperature all showed clear seasonal variation during the study period. High rainfall levels, high relative-humidity levels and low temperature levels were observed towards the end of each year (i.e. 2011 and 2012). Relatively low rainfall and relative-humidity levels and high temperature levels were observed during May–June in 2012. Monthly time series of the averaged meteorological variables are shown in Figure 1. Summary statistics are given in Table 1.

**Table 1.** Summary statistics of average rainfall, minimum temperature, maximum temperature, minimum relative humidity and maximum relative humidity in bitten months

Variable	Mean (SD)	Median (inter-quartile range)	Coefficient of variation
Average rainfall over bitten months	143 (105) mm	140 (89.5–260.5) mm	73
Average minimum temperature over bitten months	22.3 (1.3) °C	22.0 (21.5–23.0) °C	5.9
Average maximum temperature over bitten months	30.4 (1.5) °C	31.0 (30.0–31.0) °C	4.8
Average minimum relative humidity over bitten months	71.9 (4.0) %	73.0 (70.0–76.0) %	5.6
Average maximum relative humidity over bitten months	87.2 (3.4) %	89.0 (85.5–91.0) %	3.9

### Explanatory analysis

The fitted log-linear model for the recorded monthly numbers of snakebites is summarized in Table 2. Adequacy of the fitted model was assessed using the deviance statistic, a residual vs fitted value plot and an autocorrelation plot. Residual deviance of the model was 146.56 with residual degrees of freedom 147; the associated chi-squared (generalized likelihood ratio) goodness-of-fit test gave no evidence of over-dispersion relative to the assumed Poisson distribution (*p* = 0.50). Plots of standardized residuals against fitted values, and of the residual autocorrelation, also gave no evidence of lack of fit (Supplementary Figure 3A and B, available as Supplementary data at *IJE* online). Observed and predicted numbers of snakebites for each bitten month are shown in Supplementary Figure 4, available as Supplementary data at *IJE* online.

**Table 2.** Parameter estimates from log-linear model (i.e. estimates are in log scale)

Variable	Estimate	Std. error	*Z*-value	*P*-value
Intercept	9.08684	0.06680	136.03	<0.001
Cosine 12t	–0.16675	0.06139	–2.72	0.007
Sine 12t	0.04025	0.05700	0.71	0.48
Cosine 6t	–0.05925	0.05848	–1.01	0.31
Sine 6t	–0.10988	0.05669	–1.94	0.05
Cosine 4t	0.07950	0.05451	1.46	0.14
Sine 4t	0.22213	0.05911	3.76	<0.001
Cosine 3t	–0.14330	0.06153	–2.33	0.02
Sine 3t	0.08730	0.05349	1.63	0.10
Maximum relative-humidity anomaly	–0.10892	0.03474	–3.13	<0.001
Recall (months)	–0.07953	0.00954	–8.34	<0.001
Offset = log(survey effort)				

Null deviance : 273.39 on 157 degrees of freedom.

Residual deviance : 146.56 on 147 degrees of freedom.

Reported monthly snakebites showed a complex seasonal pattern, with contributions from sine–cosine pairs at 12-, 6-, 4- and 3-month periodicities. They also showed associations with maximum relative-humidity anomalies and with recall duration, i.e. the number of months between bitten month and survey month. The expected number of snakebites was higher in months with lower-than-expected average maximum relative humidity. The expected number of *reported* snakebites decreased with increasing recall duration; the estimated snakebite recall probabilities for recall durations 3, 6 and 12 months were 0.79, 0.62 and 0.39, respectively. Supplementary Figure 5, available as Supplementary data at *IJE* online, compares the expected numbers of snakebites in the sample with and without adjustment for recall bias.

### Snakebite temporal patterns

**Figure 2.**
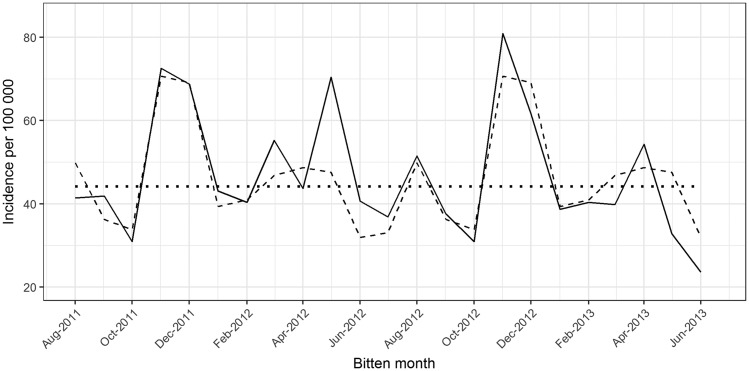
Decomposed snakebite incidence plot. Dotted line indicates the estimated snakebite incidence without seasonal and weather effects (i.e. intercept of the fitted model). Dashed line indicates the seasonal variation in snakebite incidence without the weather effect (i.e. intercept and harmonic functions of the fitted model). Solid line indicates the estimated snakebite incidence for the study period including seasonal and weather effects (i.e. full model).

**Figure 3.**
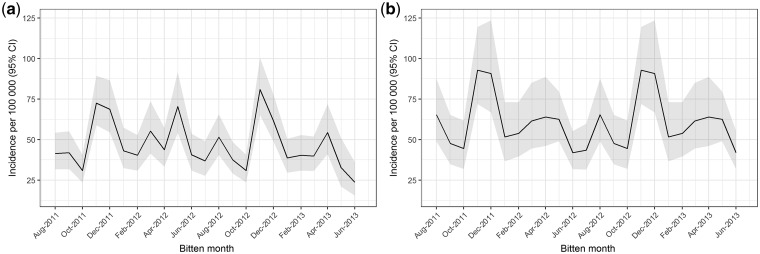
(**a**) Estimated snakebite incidence for the study period with 95% confidence interval. (**b**) Predicted snakebite incidence with 95% confidence interval for the scenario of a 2.5% reduction in maximum relative humidity, corresponding to a 0.5°C increase in maximum temperature levels holding rainfall fixed at current levels.

Snakebites showed a seasonal pattern and an independent association with maximum relative-humidity anomaly (Figure 2). According to the pure snakebite seasonal pattern, the highest expected snakebite incidence occurs during November–December, followed by March–May and August, whilst the lowest occurs during June–July, followed by October. The highest and lowest respective snakebite incidences are 70.7 (95% CI: 57.3–87.2) and 31.9 (95% CI: 23.5–43.3) per 100 000, whilst the overall monthly incidence is 45.7 (95% CI: 35.4–59.0). Lower-than-expected maximum humidity levels contribute to an increase in snakebite incidence and vice versa. For instance, expected snakebite incidences for May 2012 and May 2013 are 70.4 (95% CI: 54.2–91.5) and 32.9 (95% CI: 21.1–51.2) per 100 000, respectively, due to the differences in relative-humidity levels (i.e. –3.588 and 3.404, respectively) in these 2 months (Figure 3a).

Our model predicts the total number of snakebites in Sri Lanka during the calendar year 2012 to have been 119 000 (95% CI: 103 000–134 000) after accounting for recall bias and survey effort. Had there been no deviations from expectation in the seasonal weather patterns, i.e. zero anomaly in the maximum relative-humidity levels, during 2012, the predicted number would have been 110 000 (95% CI: 95 000–124 000).

### Effects of different climate-change scenarios

According to our model, a 1% reduction in maximum relative humidity at all times of the year holding rainfall fixed at current levels will increase snakebites by an estimated 11.5% (95% CI: 4.1–19.4). Similarly, a 2.5% reduction in maximum relative humidity, corresponding to a 0.5°C increase in maximum temperature levels, will increase snakebites by 31.3% (95% CI: 10.7–55.7). Under the projection of a 0.5°C increase in maximum temperature levels nationwide, these results imply the following. First, the expected annual snakebite burden will increase to 144 000 (95% CI: 122 000–166 000). Second, the incidence per 100 000 will rise to 92.8 (95% CI: 72.0–119.6) in November, the month of highest risk, and to 41.9 (95% CI: 31.8–55.3) in June, the month of lowest incidence. This will result in an overall monthly incidence of 60.0 (95% CI: 44.5–81.0) (Figure 3b).

An alternative scenario is that a temperature increase associated with climate change will affect primarily dry months. A 1°C temperature increase over 6 dry months together with no change in the other 6 months will increase the overall monthly incidence to 62.2 (95% CI: 49.1–74.1) per 100 000 and total number of snakebites to 147 000 (95% CI: 120 000–1 740 000) nationwide.

In summary, a 0.5°C average increase in temperature will substantially increase snakebites in Sri Lanka to a similar extent whether this average temperature increase is spread over the whole year or concentrated into the 6 dry months.

## Discussion

The National Snakebite Survey showed clear seasonal variation in snakebite incidence. Snakebites showed a complex seasonal pattern independently of the seasonal weather anomalies. The highest snakebite incidence was observed during the period November–December, with smaller peaks in March–May and August. Under average weather conditions, Sri Lanka can expect monthly snakebite incidence of 46 bites per 100 000 population per month on average, increasing to 71 bites per 100 000 during November–December. Periods of lower-than-expected relative-humidity levels lead to higher-than-expected snakebite incidence and vice versa. Our estimated seasonal snakebite peaks are compatible with previous local data.^6^^,^^7^^,^^16^ Similar seasonal patterns have also been observed in other countries in the region.^9^^,^^29–32^

Regular seasonal weather patterns (i.e. rainfall, temperature and humidity) are closely associated with rotational movement of the Earth around the Sun. We therefore used harmonic mathematical functions to represent annual, biannual, quarterly and three-monthly regular components of variation in incidence. All the variables representing weather conditions were considered for inclusion in the model as anomalies, i.e. departures from expectation for the month in question, again using harmonic mathematical functions to describe their expected patterns of seasonal variation. This use of anomalies, rather than actual values, of weather-related variables enabled us to evaluate the effects of weather conditions independently of other seasonally varying factors with which they would otherwise have been confounded.^33^^,^^34^ Therefore, the purely seasonal effects in the model (sine and cosine terms) can be considered as proxies for seasonally varying *expected* weather and agricultural patterns, and *weather anomaly* effects as weather effects that cannot be explained by the purely seasonal pattern. After accounting for the purely seasonal weather pattern, snakebites showed association with maximum ambient relative humidity, but not with rainfall or temperature.^35^ Monthly average maximum relative humidity showed the lowest coefficient of variation among the meteorological variables and exhibited more pronounced changes over time than either temperature or rainfall. Thus, maximum relative humidity can be considered as a better representative of relevant monthly weather conditions in our study. A positive correlation between rainfall and snakebite has been shown in a number of studies,^36–38^ but it is possible that these reported correlations could be due to the rainfall acting as a proxy for the combined effects of unmeasured social and environmental factors.

Lower-than-expected maximum humidity levels in a given month were associated with higher numbers of snakebites and vice versa. This was clearly seen, e.g., in a comparison between May 2012 and May 2013, where estimated incidence was higher and lower, respectively, than the expected incidence according to the pure seasonal pattern. Previous literature has shown that certain snake species can be encountered more frequently on lower humid days and dry seasons, and high snake activity patterns may predispose to snakebites.^39^^,^^40^ During dry periods, Sri Lanka experiences high temperature and low rainfall, leading to low relative humid environmental conditions. More snakebites can be expected during such dry periods. A similar observation has been made in Costa Rica (Latin America), where more snakebites have been recorded in high-temperature and low-rainfall conditions.^24^

Snakebite patterns coincide with the agricultural seasons, with high numbers of snakebites observed at the beginning and end of the agricultural seasons corresponding to cultivation and harvesting times. The ‘Maha’ season is the main agricultural season in the country, starting during November–December. This explains the associated high snakebite burden during this time compared with the much lower incidence in the ‘Yala’ season that starts around May, when agricultural activities are largely confined to areas fed with irrigated water. On the other hand, heavy physical farming activities, leading to greater human interaction with the environment, are usually over before the onset of heavy rains during the agricultural seasons and a correspondingly low snakebite incidence can be observed during the mid-seasons. Subsequently, harvesting starts after the heavy rains and high snakebite incidence can again be observed towards the end of farming seasons.^6^^,^^7^^,^^16^ Lower relative-humidity levels are expected at the beginning and end of each agricultural season compared with mid-season when rainfall is generally heaviest, and this is reflected in the estimated snakebite pattern during an agricultural season. In 1978, the Accelerated Mahaweli Programme deforested nearly 130 000 hectares of land in the country and settled farming communities in the dry zone. Subsequently, rodents, lizards and other potential snake-prey populations increased near human dwellings, especially during the harvesting seasons, predisposing to snakebites.^29^^,^^41^ Also, outdoor activities take place during the dry warmer months, leading to high snakebite incidence during such months.^42^^,^^43^

Global climate-change models have projected that Sri Lanka will experience an increase of about 1.0–2.0°C in its maximum temperature levels by 2100 under the RCP 4.5 and RCP 6 scenarios. Accordingly, we can expect a 0.5°C increase in maximum temperature levels over the next 25–50 years.^20^ We considered the inverse physical relationship between temperature and relative humidity in order to make future projections of snakebite incidence due to the global climate change.^28^ According to the fitted model, snakebite burden will escalate along with the increasing maximum temperature levels, and we should expect a nationwide increase of snakebites by 31% per calendar year over the next 25–50 years. Either homogenous or heterogeneous temperature increases over a year under global climate change will increase the national burden of snakebite to a similar extent. Increasing temperature will cause expansion of dry seasons and the drying-up of water sources, tanks and rivers. These changes affect snake ecosystems and the associated reduction in humidity levels will lead to further increase in snakebites.^22^ Hot weather conditions encourage foraging activity in reptiles^8^^,^^44^^,^^45^ and lack of water and food forces both snakes and snake-prey into human habitats, leading to an increase in the numbers of accidental snakebites.^46^ Therefore, it is important to identify snakebite as a health priority in a time of global climate change.^22^^,^^24^

Accuracy of recall of an event in an epidemiological study is related to the characteristics of the respondent and of the event of interest.^47^ Although snakebite events are significant events, still it is possible that victims and family members will forget the event. The National Snakebite Survey captured the snakebite events that occurred in the preceding year, and it could be anticipated that recorded bitten numbers will suffer from recall bias. We addressed this in our analysis by an exponentially decaying function of recall duration. Our analysis showed a significant and substantial recall effect, and our final monthly snakebite estimates were derived after correcting for this. A particular consequence of recall bias is that the true burden of snakebite in Sri Lanka is substantially greater than would be deduced simply from the number of snakebites recorded during the survey. This explains why a previous report based on a purely spatial analysis^1^ gave the estimated total number of snakebites occurring in Sri Lanka as 80 000 bites per year, whereas the present analysis that accounts for recall bias estimates the annual snakebite burden to be approximately 110 000 under expected weather conditions, rising to 120 000 in years such as 2012, when humidity is lower than expectations.

The National Snakebite Survey was conducted over 11 months. Survey effort varied considerably from month to month due to the variation in population density in the surveyed areas as well as the geographical extent surveyed during a month. Similarly, the effective sample size for each bitten month varied from month to month. These features of the survey methodology were addressed by considering the number of bites pertaining to each combination of bitten month and survey month as the response variable in a log-linear model that included an offset to represent the fraction of population sampled during each survey month. This allowed us to estimate the risk of snakebites at the national level in each bitten month.

### Limitations

Our estimates are limited to inference on variation in snakebite incidence over a relatively short period of 23 months. Average monthly dew point would have been a better indicator of variation in monthly humidity than the average relative humidity, but the relevant data could not be obtained for the analysis. Our predictions of the effect of increasing temperature in snakebite incidence used the physical relationship between temperature and relative humidity, and the change in relative-humidity level resulting from an increase in temperature was calculated assuming other environmental conditions are fixed. In other words, we estimated the effect of global climate change based purely on a change in expected temperature and no change to rainfall. However, climate-change effects can only be explained fully by considering changes in ecosystems, population growth, land use, urbanization and access to food and water.^48^ The aim of the present study has been to evaluate temporal variation in snakebite burden at the national level. We already know that snakebite burden exhibits spatial variation.^1^ Our preliminary investigations suggest that local patterns of temporal variation in snakebite burden also vary across the country. A likely contributory factor to this is that monsoon seasons occur at different times of the year in the different parts of the country. We intend to investigate this, and other aspects of spatio-temporal interaction, in a future study.

The present study has aimed only to evaluate snakebite burden at the national level. Seasonal patterns may differ at the regional level and it is known that different snake species respond differently to changes in weather conditions.^24^^,^^35^^,^^49^ Therefore, further studies are required to evaluate spatio-temporal variation of snakebites in Sri Lanka.

## Conclusion

In conclusion, we have shown seasonal variation in snakebite incidence in Sri Lanka, an independent adverse effect of low relative-humidity level and a potential increase in snakebite burden due to changes in global climate. These findings can inform healthcare decision-making at the national level. Our methodology for addressing recall bias and month-to-month variation in survey effort can be implemented in any generalized linear modelling software and is applicable to any epidemiological survey in which these features appear.

## Funding 

National Snakebite Survey was supported by the National Health Medical Research Council, Australia [NHMRC 631073, NHMRC 630650, NHMRC Program Grant 1055176, NHMRC Practitioner Fellowship 1059542, NHMRC Senior Research Fellowship 1061041]. Dileepa Ediriweera is supported by the Medical Research Council [MR/P024513/1].

## Supplementary Material

Supplementary Figure S1

Supplementary Figure S2

Supplementary Figure S3

Supplementary Figure S4

Supplementary Figure S5

Supplementary Material
