# Supplementary material for: Evaluating temporal patterns of snakebite in Sri Lanka: the potential for higher snakebite burdens with climate change
Source: Int J Epidemiol. 2018 Sep 11;47(6):2049–58. doi: 10.1093/ije/dyy188 (PMC6280932; doi:10.1093/ije/dyy188)
Supplement: Supplementary Figure S2 [file dyy188_supplementary_figure_s2.docx]

# Supplementary Material

## Figure S2


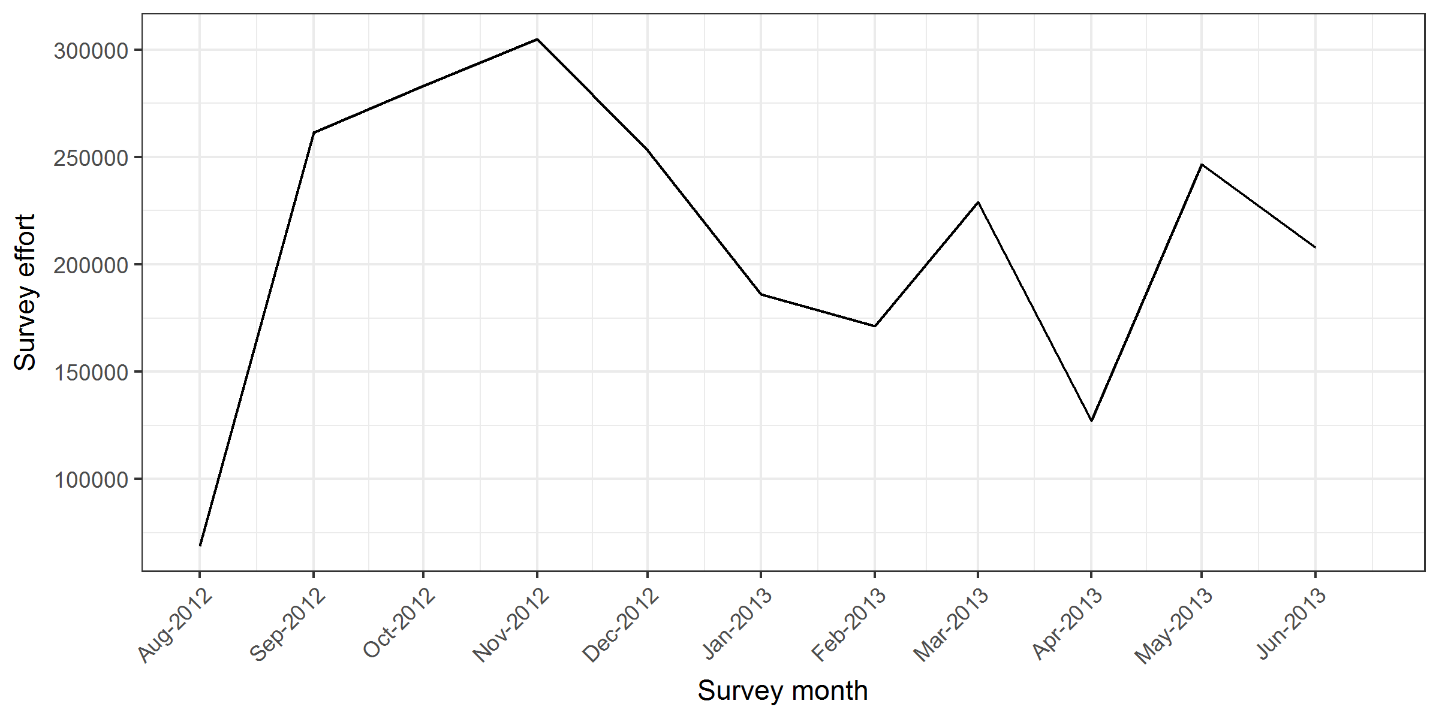


Figure S2: Variation of survey effort (i.e. number of people surveyed) during the survey period.
