# Supplementary material for: Evaluating temporal patterns of snakebite in Sri Lanka: the potential for higher snakebite burdens with climate change
Source: Int J Epidemiol. 2018 Sep 11;47(6):2049–58. doi: 10.1093/ije/dyy188 (PMC6280932; doi:10.1093/ije/dyy188)
Supplement: Supplementary Figure S3 [file dyy188_supplementary_figure_s3.docx]

# Supplementary Material

## Figure S3:

| 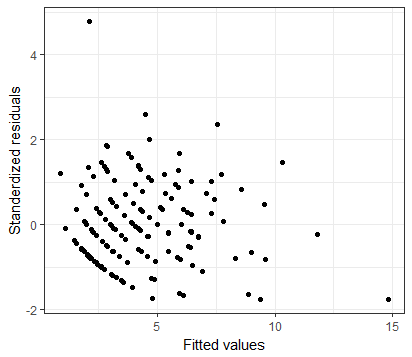 | 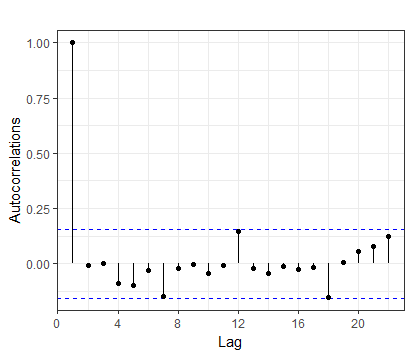 |
| --- | --- |

Figure S3: Residual plots of fitted model: (a) Standardized residuals against fitted values. (b) Autocorrelation of standardized residuals.
