# Supplementary material for: Evaluating temporal patterns of snakebite in Sri Lanka: the potential for higher snakebite burdens with climate change
Source: Int J Epidemiol. 2018 Sep 11;47(6):2049–58. doi: 10.1093/ije/dyy188 (PMC6280932; doi:10.1093/ije/dyy188)
Supplement: Supplementary Figure S4 [file dyy188_supplementary_figure_s4.docx]

# Supplementary Material

## Figure S4:


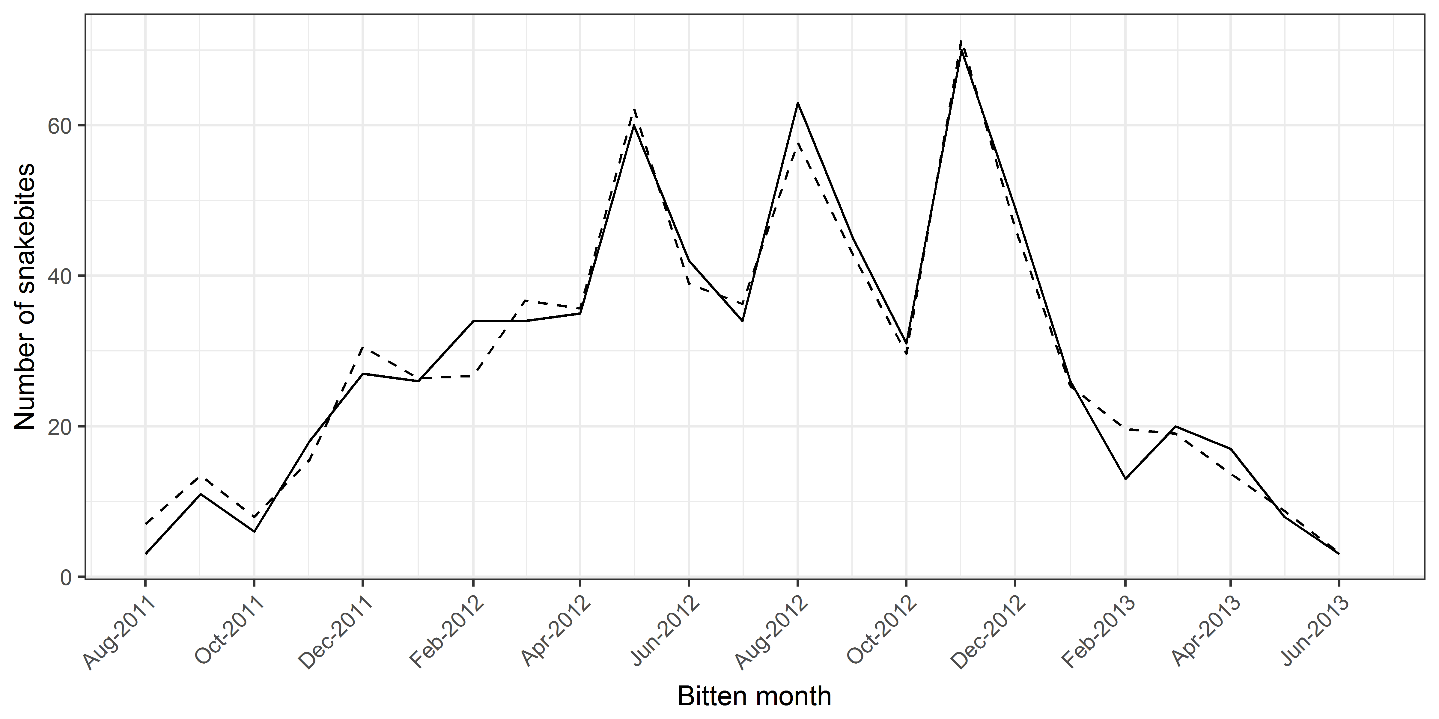


Figure S4: Number of observed snakebites in the survey (solid line) and the number of estimated snakebites from the fitted model (dashed line) for each bitten month. The solid line indicates the total number of observed snakebites in the survey by disaggregating into bitten month without adjusting for survey effort or recall bias.
