# Supplementary material for: Evaluating temporal patterns of snakebite in Sri Lanka: the potential for higher snakebite burdens with climate change
Source: Int J Epidemiol. 2018 Sep 11;47(6):2049–58. doi: 10.1093/ije/dyy188 (PMC6280932; doi:10.1093/ije/dyy188)
Supplement: Supplementary Material [file dyy188_supplementary_material.docx]

# Supplementary Material

## Bootstrap validation of confidence intervals

All confidence intervals quoted in the paper rely on the standard assumption that maximum likelihood estimates are approximately unbiased and Normally distributed. To check the accuracy of this assumption, we also used the following bootstrap method. We drew 10,000 samples with replacement from the survey data sample. For each such sample, we obtained parameter estimates and used these to compute estimates of any relevant function of the parameters, specifically the total number of bites per calendar year. The interval between the 2.5^th^ and 97.5^th^ centiles of the 10,000 estimates then constitutes a bootstrapped 95% confidence interval. In all cases, the standard and bootstrapped confidence intervals were in close agreement.

Table S1. Standard and bootstrapped 95% confidence interval for total annual numbers of snakebites under two scenarios

| Scenario | Standard | Bootstrap |
| --- | --- | --- |
| Current | 118,596 (102,955 – 134,237) | 119,546 (102,709 – 138,221) |
| 0.5C increase in temperature | 144,008 (121,953 – 166,063) | 145,406 (116 971 -177,677) |
